# Advancing Precambrian palaeomagnetism with the PALEOMAGIA and PINT(_QPI_) databases

**DOI:** 10.1038/sdata.2017.68

**Published:** 2017-05-23

**Authors:** Toni H. Veikkolainen, Andrew J. Biggin, Lauri J. Pesonen, David A. Evans, Nicholas A. Jarboe

**Affiliations:** 1 Department of Physics, Division of Materials Physics, University of Helsinki, PB 64, FI-00014 Helsinki, Finland; 2 Geomagnetism Laboratory, Oliver Lodge Building, Department of Earth, Ocean and Ecological Sciences, University of Liverpool, Liverpool L697ZE, UK; 3 Department of Geology and Geophysics, Yale University, PB 208109, New Haven, Connecticut 06511, USA; 4 Scripps Institution of Oceanography, University of California, San Diego, 8635 Kennel Way, La Jolla, California 92093-0220, USA

**Keywords:** Palaeomagnetism, Geomagnetism

## Abstract

State-of-the-art measurements of the direction and intensity of Earth’s ancient magnetic field have made important contributions to our understanding of the geology and palaeogeography of Precambrian Earth. The PALEOMAGIA and PINT(_QPI_) databases provide thorough public collections of important palaeomagnetic data of this kind. They comprise more than 4,100 observations in total and have been essential in supporting our international collaborative efforts to understand Earth's magnetic history on a timescale far longer than that of the present Phanerozoic Eon. Here, we provide an overview of the technical structure and applications of both databases, paying particular attention to recent improvements and discoveries.

## Background & summary

The Precambrian, 540–4,567 million years ago (Ma), spans nearly 90% of geological history and yet far less is understood about its geological processes, shallow and deep, than about the subsequent Phanerozoic Eon. Knowledge of the Precambrian geology owes much to palaeomagnetism, the study of the direction and intensity of the Earth’s ancient magnetic field. Palaeomagnetic information is preserved in ferromagnetic minerals of rocks and sediments and it can be discovered after demagnetization, the removal of the present day field vector from samples in laboratory conditions to reveal the original thermoremanent magnetization (thermoremanence; TRM). Most importantly, palaeomagnetism has been largely used to reconstruct past positions of continents and it has advanced our understanding of the supercontinent cycle^1,2^.

Palaeomagnetic information has mostly been published as directional data, using declination-inclination and palaeomagnetic pole latitude-longitude pairs. The International Association of Geomagnetism and Aeronomy’s (IAGA) Global Paleomagnetic Database has been one of the most important contributions of this kind (http://www.ngdc.noaa.gov/geomag/paleo.shtml), yet it has been left unattended since December 2004. Researchers have thereafterintroduced new databases for particular periods, such as the archaeomagnetic database GEOMAGIA^3,4^ and the Magnetics Information Consortium database^5^ (MagIC, https://earthref.org/MagIC).

The Palaeomagnetic Information Archive (PALEOMAGIA), which hosts only Precambrian data, aimed to answer the call for an up-to-date and easily accessible resource. The first version was released in 2014 (ref. 6), and development of the database has been continuous thereafter. PALEOMAGIA now contains as many as 3,494 directional data associated with 1,013 individual palaeomagnetic studies and 608 age references, thus providing an unparalleled platform for researchers of Precambrian plate motions and supercontinents. The database also features a contemporary and intuitive user interface with a map of sampling locations and links to original publications whenever available. In addition, referenced isotopic age information added to PALEOMAGIA in 2015–2017 significantly facilitates the construction of supercontinent models.

Without palaeomagnetic data, plate tectonic models of the far past can only be constructed qualitatively, e.g., by using ancient mountain belt successions from one continent to another. For example, researchers have used only geological piercing points to suggest Proterozoic connections between the Southwestern United States and East Antarctica, as well as Australia and the Western United States^7,8^. Quantitative palaeomagnetic results have rendered these hypotheses questionable^9^. Although purely geological continent assemblies, such as the South America –Eastern Europe connection, are still being suggested^10^, their validity should be reviewed in light of well-established primary palaeomagnetic poles from the same continents. A series of palaeomagnetic poles of different ages from a certain continent forms an apparent polar wander path (APWP). Ideally, if APWPs of two continents or more have similar characteristics, the continents have shared a common history. Introduced in 1954 (ref. 11), the concept of APWPs has been crucial to a number of paleomagnetically viable supercontinent models, Precambrian and Phanerozoic alike.

In contrast, estimates of ancient magnetic intensities (palaeointensity) have proven invaluable for constraining the thermal evolution of the deep Earth^12,13^. For these data, we have also established a separate database called PINT (http://earth.liv.ac.uk/pint/). We have recently assigned a comprehensive quality metric (Q_PI_ ) to all Precambrian palaeointensity values in PINT, alongside recent additions from rocks of all ages^14^ (http://qpi.wikispaces.com/). These criteria are reminiscent of the directional quality grading^15^ but always applied at a site mean level. In addition to the eight criteria presented by ref. 14, a recent study^12^ introduced a new one to indicate whether the raw measurement data are publicly available, e.g., in MagIC (Table 1). The new dataset, referred to as PINT(_QPI_), follows the arrangement of the original PINT database, and incorporates: age, location, and lithology of the palaeointensity measurements; site-level palaeomagnetic directional information; and measurement details such as experiment type, number of specimens and standard deviation used for the calculation of the virtual axial dipole moment. A.J.B., T.H.V., L.J.P. and co-authors recently used the PINT(_QPI_) database to support a hypothesis that the inner core formed between 1,000 and 1,500 Ma ref. 12), whereas T.H.V., L.J.P. and D.A.E. used PALEOMAGIA data to further support works that have argued for a geocentric dipole in the Precambrian^16,17^. The debate, however, over the inner core age still continues^18^.

## Methods

### PALEOMAGIA

In the first phase of the construction of PALEOMAGIA (Data Citation 1), T.H.V. and L.J.P. imported preformatted text files from version 4.6 of the IAGA Global Paleomagnetic Database to Microsoft Excel tables, and leading scientists working in the field of palaeomagnetism checked the data. Thereafter numerous data were added from other sources, mostly from peer-reviewed journals but also from monographs, doctoral theses and national geological survey reports.

**Figure 1:**
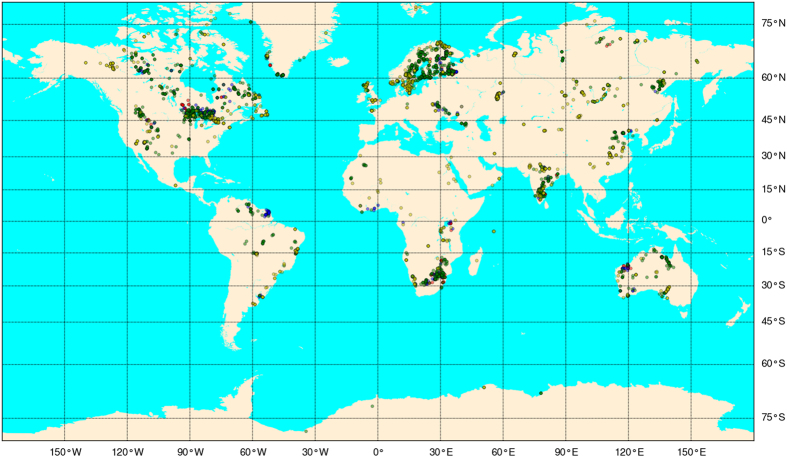
PALEOMAGIA sampling locations on a world map. Symbols are semi-transparent and a darker colour therefore indicates a greater number of data at a given site. Red symbols refer to Archaean (*N*=191), blue symbols to Palaeoproterozoic (*N*=438), green symbols to Mesoproterozoic (*N*=1,953) and yellow symbols to Neoproterozoic (*N*=912) data. Miller projection.

The online PALEOMAGIA database^6^ first opened in November 2013 on a server at the University of Helsinki, Finland, with a structure based on one table for each continent. This was later replaced by a website with a true relational database and a host of additional features, such as a dynamically generated list of all poles published, a table with information about all terranes in the database, a comprehensive age reference list and also a Google map featuring all sampling sites. In the map, polygons delineate schematic Precambrian borders for Laurentia-Greenland, Amazonia-Guyana, Baltica, India, Kaapvaal, North China, Pilbara, Siberia, South China and Yilgarn. PALEOMAGIA currently contains 3,494 directional data records, yet the spatial distribution is highly uneven (Fig. 1).

The PALEOMAGIA website features a query form, which supports the selection or deselection of peer-reviewed and non-reviewed data and subselection on the basis of three major rock types (igneous, sedimentary and crystalline rocks). Location-based filtering can be performed simultaneously either geographically, using borders of present-day countries, or geologically, using certain large, well defined Precambrian continents (e.g., Baltica, Laurentia) or smaller units such as cratons, orogens and inliers. These smaller units are referred to in the database as terranes. Some of them, e.g., Tarim and Rio de la Plata, are fragments, which do not belong to any specific Precambrian continent. The other ones are related to continents in the query form in a manner illustrated by the following examples:

- BALTICA (ALL): Baltica-Ukraine, Baltica-Ural, Baltica-Rest
- INDIA (ALL): India-Bastar, India-Bundelkhand, India-Dharwar, India—Eastern Ghats, India-Rajasthan, India-Singhbhum, India—Tamil Nadu

In the above cases, all terranes beginning with name ‘Baltica’ or ‘India’ are taken into account, as they were parts of the larger continent in a certain timeslot. For instance, Bundelkhand craton was a part of Indian continent for the entire Precambrian timespan with palaeomagnetic data available. On the contrary, poles of the Archaean- Early Mesoproterozoic (3.0–1.8 billion years) Karelia and Kola cratons are not considered in the query, despite the fact that their poles partly populate the same present-day area as poles of unified Baltica, which amalgamated later, at 1.7–1.8 billion years (Ga)^19^.

A PALEOMAGIA user may select from a variety of search options. The result page of the database also allows users to go back and change only the desired criteria, instead of requiring them to reselect everything afresh.

### PINT(_QPI_)

PINT(_QPI_) (Data Citation 1) is an expanded version of the PINT database of published Palaeomagnetic field INTensity estimates, which was initiated in 1987 under the auspices of the International Association of Geomagnetism and Aeronomy. PINT has been under the management of several different individuals and has undergone numerous iterations that are described in detail elsewhere^20–22^. It is currently accessible via a queryable interface hosted by the University of Liverpool (http://earth.liv.ac.uk/pint/) where, since the advent of MagIC, it no longer falls under the remit of IAGA. Full details regarding the structure of, and recent updates to, PINT can be viewed at this website.

PINT undergoes updates once per year on average whereby newly published, or newly discovered, peer-reviewed publications containing palaeointensity estimates are collated from internet searches and mined for new records. PINT(_QPI_) is the 2015.05 version of PINT, only including records, which have been deliberately assessed for the purpose of calculating the Q_PI_ value, which aims to provide an objective and useful indicator of reliability^14^.

Since assigning Q_PI_ values consistently is a time-consuming process, PINT(_QPI_) only contains 15% (642 records) of the 4,293 records in PINT; however, this includes all records dated to older than 500 million years. All future updates of PINT will include an assignment of the Q_PI_ value and work has already begun on assigning values retrospectively; this percentage is therefore expected to increase in future versions and eventually PINT(_QPI_) will replace PINT altogether, being an online queryable database rather than just a data compilation.

### Code availability

The PALEOMAGIA user interface has been built using HTML 4.01 and CSS 3.0. Server-side scripting, including but not limited to scripts connecting to the MySQL database, employs PHP 5.3.3. Unlike static code, these scripts do not appear to the database user online since they are run at the server prior to the loading of the page in the browser. Client-side scripting, such as the management of submission buttons, sorting of tables and connections to Google Maps, has been done using Javascript. The source code is available from T.H.V. via email upon request (toni.veikkolainen@helsinki.fi).

The PALEOMAGIA database employs the MariaDB MySQL standard and can be easily edited in online database management tools such as phpMyAdmin (http://www.phpmyadmin.net/). In addition, a static copy of the database tables (PALEOMAGIA 2.00, as of July 15, 2016) is freely available in the Open Document spreadsheet format (ods) at Dryad for reuse under the CC0 waiver (Data Citation 1). However, since data evolve with time, users are advised to access the most recent version at http://www.helsinki.fi/paleomagia (PALEOMAGIA 2.03, as of March 9, 2017). The original code used to generate the location map available at the database website follows Google Maps API, which can be used under the conditions issued by Google (https://developers.google.com/maps/).

While PINT is accessible through a web-based (ASP.NET) queryable interface linked to an MS Access database, PINT(_QPI_) is presently only available as a spreadsheet. This is available in MS Excel format at a dedicated Wikispaces site (http://qpi.wikispaces.com/), which also provides a forum for suggesting changes to the Q_PI_ values. A copy of the spreadsheet as of July 15, 2016 in the Open Document spreadsheet format is also available at Dryad in the same package with PALEOMAGIA files (Data Citation 1), although this will not be updated.

## Data Records

PALEOMAGIA is a relational database (Tables 2,3 (available online only), 4) where each palaeomagnetic record in the main data table (*data*) is related to references in separate tables: one for its age constraints (*agerefs*) and another for its palaeomagnetic directional information *(pmagrefs).* Country and terrane name information is stored in tables named *countries* and *terranes*. The main data table is also related to these two tables. The output of the database query page combines information from all database tables selectively, and adds certain useful quantities, which are calculated dynamically using server-side scripts. For example, palaeolatitudes (λ) are related to inclinations (I) via tan I=2 tan λ^23^ and therefore palaeolatitude data are not directly stored in the database structure. The terrane configuration follows both present-day geography and Precambrian geology, which has evolved with time, and therefore users are advised to check the geological age range associated with each terrane from the database website.

**Figure 2:**
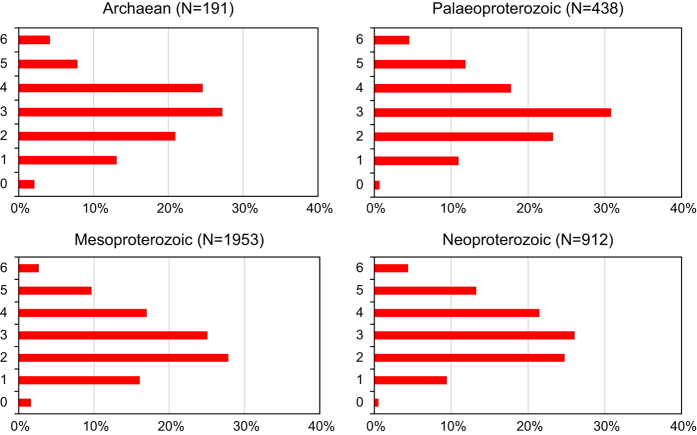
PALEOMAGIA data grouped by geological age and the sum of Q_Voo_ criteria (Table 3 (available online only)) in each group, excluding the seventh criterion as explained in the text.

The majority of numeric information in PALEOMAGIA (Data Citation 1) has been input as published because the database relies mostly on peer-reviewed data, which do not require additional validation. Before being added to the database, however, all entries are assigned directional quality ratings^15^ with the exception that the seventh criterion, which refers to the pole not resembling any younger pole, is not considered. This truncated Q_Voo_ quality scale has previously been employed, for example, in refs 16 and 24 because use of the full seven-grade rating would necessitate knowledge of precise Phanerozoic APWPs for most continents. Unfortunately, the Precambrian palaeomagnetic community currently does not have this information, which is somewhat subjective and can change as new data arise. For further reasons for the truncation of the scale, the reader is referred to ref. 6. A comparison of Q_Voo_ values for Archaean, Palaeo-, Meso- and Neoproterozoic PALEOMAGIA data (2,500–4,567 Ma; 2,000–2,499 Ma; 1,000–1,999 Ma; 540–999 Ma; Fig. 2) shows that there is no substantial dependence of the quality of data on the geological age. Reporting Q_Voo_ values, either full or truncated, has long been standard practice in the palaeomagnetic literature and has led to an overall increase in the quality of data^6^.

To make PALEOMAGIA as comprehensive as possible, the database management team works to find new data with the aid of alert services provided by major science publishers. The palaeomagnetic community can also suggest their own publications to be included, using the specific data suggestion form at the website. Database administrators, however, reserve the right to accept or reject any suggestion based on the relevance of data suggested. For example, data from non-reviewed conference proceedings are not considered in a case where similar data have been published in a peer-reviewed journal. No article copies are stored on database servers. For PINT, suggestions of new data should be addressed directly to A.J.B. via email (biggin@liverpool.ac.uk) or posted as a comment to the forum page of QPI Wiki.

**Figure 3:**
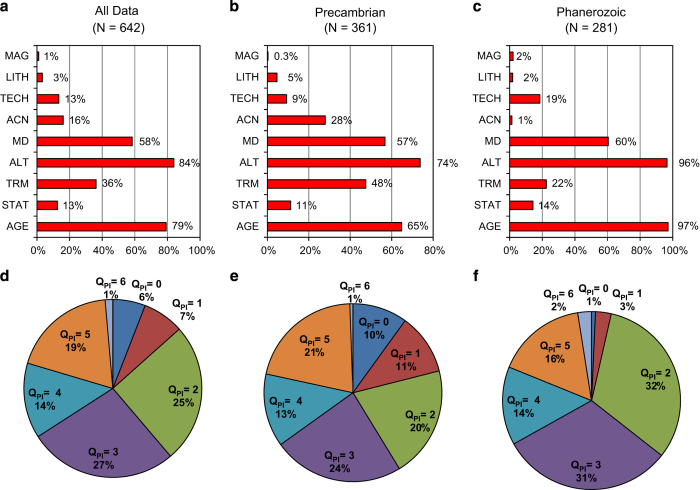
Assignment of Q_PI_ criteria and overall values within PINT(_QPI_).All data are shown in (a) and (d), Precambrian only in (b) and (e) and Phanerozoic only in (c) and (f). Although the current edition of PINT(_QPI_) incorporates only 642 out of 4,293 data entries in PINT, progress is being made to extend the Phanerozoic part of PINT(_QPI_).

The PINT(_QPI_) database (Data Citation 1) contains four tables (Table 5) presented as worksheets in the spreadsheet: one each for the *Data* and *Ref* tables (linked by the REFNO URN); one containing a legend of the palaeointensity techniques (*PIMethods*); and one containing explanations of the fields in the *Data* table (*Information*). Figure 3 reports the percentages of palaeointensity estimates meeting individual Q_PI_ criteria and assigned different Q_PI_ values, broken down into age groups of Precambrian (540 Ma and older) and Phanerozoic (younger than 540 Ma). The most common criterion to be met in both groups is ALT, which reflects the tendency of most modern palaeointensity studies to incorporate checks for sample alteration in the laboratory. The least fulfilled criterion is MAG, which reflects the availability of raw specimen level data associated with the palaeointensity estimate. By this, we refer to full-vector magnetization or moment data produced at each measurement step alongside appropriate metadata concerning the individual treatment, e.g., peak temperature, applied magnetic field vector, etc. This reflects a community desire for access to data at a level whereby they can be reanalyzed, potentially offering new insight when large database-wide studies are undertaken. This reporting of data has become a requirement of many funding agencies over the last two years, and MagIC is a primary community database for palaeomagnetic and palaeointensity data.

## Technical Validation

Up-to-date and reliable age information is essential and fully referenced isotopic ages have been used in PALEOMAGIA, whenever they have been available and considered credible. Entries lacking isotopic age constraints are typically assigned ages based on APWPs, correlation to other similar units or stratigraphy. The PALEOMAGIA documentation provides letter codes for various age determination methods as well as other useful information for database users such as abbreviations of lengthy journal names, which are used to save space (http://www.helsinki.fi/paleomagia/documentation.php). Whenever new data prove that a database entry that was formerly considered Precambrian is actually Phanerozoic, PALEOMAGIA administrators move it to a reserve database, where it is no longer visible to the public but can be pulled back to the public database if additional evidence of its Precambrian age is published. In the case of some poles, such as Proterozoic moderate- and high-inclination data of Baltica, e.g., Salla dykes^25^, concerns about the age of magnetization are addressed in the comment section of the data table, yet the choice of inclusion or exclusion of the pole in an analysis is left to the database user. In fact, a number of Baltic Shield poles formerly regarded as Precambrian may actually be Jurassic overprints^26^, another recent finding based on PALEOMAGIA.

The validation of magnetic polarities for Precambrian data is more difficult since APWPs for most continents and cratons, even if available, are highly uncertain and full of temporal gaps^27^. Their agreement with Phanerozoic APWPs has been also a matter of debate, despite recent promising findings based on PALEOMAGIA data of Baltic craton^24^. Both polarities should be equally represented in the palaeomagnetic record of long timescales^28,29^, but a recent comparison of Proterozoic and Phanerozoic data imply that Proterozoic observations are biased towards normal polarity, with the ratio being 57 to 43%^24^. In PALEOMAGIA, dual polarity data, e.g., Satakunta dykes^30^ are typically represented by three entries: one for normal polarity, one for reversed polarity, and one for a combination of them. No combined direction or pole has been included in PALEOMAGIA in the case where N and R data are demonstrably of different ages. This applies e.g., to the Marathon dykes, where R polarity is 2,106 Ma and N polarity 2,124 Ma^31,32^.

While the Precambrian palaeomagnetic community has mixed opinions on whether only the most reliable poles, referred to as ‘key poles’^33^ should be used in palaeogeographic studies, the application of database-wide quality criteria leaves the user the decision on sufficient quality of data to be selected. A simple dichotomy between key poles and non-key poles would not give this kind of flexibility. It is also evidentthat several key poles are composite poles gathered from a variety of studies from rocks of the same area and therefore have more than one reference, whereas PALEOMAGIA aims to keep poles from separate studies distinct, with just a few reasonable exceptions such as Bahia coastal dykes^34^ and Umkondo Large Igneous Province^35^. However, even in these situations, the authors of the original publication have sampled new data, they have not only combined data from previous sources as done e.g., in certain key poles mentioned in ref. 33.

The validation of PALEOMAGIA data has occasionally been based on personal communications, e.g., in the case of Ukrainian intrusions^36^ where the published paper did not fill the minimum criteria of palaeomagnetic information to be directly accepted to the database. The PALEOMAGIA administrators also encourage the user community to submit feedback so that possible drawbacks and inaccuracies can be addressed. The database user should view the functionality of PALEOMAGIA data in reconstructions by checking Euler rotation parameters from external sources (e.g., ref. 37).

All papers are carefully checked before palaeointensity data are appended to PINT and PINT(_QPI_). However, the question of whether estimates pass some of the Q_PI_ criteria is unavoidably subjective and therefore some effort is necessary to ensure consistency between different assessors. The manager of the database and lead author of the Q_PI_ reference paper^14^ achieves this by checking all assessments and associated notes prior to updating the database. In addition, a wiki exists for peer discussion of published Q_PI_ values and this has already been used to adjust the Q_PI_ value of one estimate based on additional information provided by the original authors. In general, we are extremely keen to promote further discussion and/or contributions to Q_PI_ assessment, including self-assessment with the original publication (e.g., ref. 38).

## Usage Notes

The PALEOMAGIA and PINT(_QPI_) data have been gathered from original publications. In certain rare cases, we have performed recalculations, especially where new age or geochemical information have required the PALEOMAGIA administrators to omit some directional data used in the original publication. These recalculations are addressed in a separate column in the database structure and are typically also mentioned in the comment field as an option to view from the query form. Recalculated data currently correspond to 125 entries, 3.6% of the entire PALEOMAGIA.

In a typical palaeomagnetic study, the sampling site location and declination-inclination pair have been regarded as primary information, whereas palaeomagnetic poles are derived information. However, in a few cases, the PALEOMAGIA administrators have needed to solve the mean location from the published direction and pole if directional information has been unavailable. Due to new information becoming available, the polarity selection in PALEOMAGIA may be different from that applied in the original publication, and this distinction is not separately addressed. Since not all palaeomagnetic quantities are recorded in all studies, the PALEOMAGIA administrators have in some cases filled missing information to the database using standard palaeomagnetic formulae (e.g., ref. 23).

In contrast with the MagIC database, PALEOMAGIA is a lightweight system intended for fast and convenient bulk data downloads in various formats, such as HTML (appearing on the website), CSV (downloadable) and XML (appearing on the website). Therefore PALEOMAGIA does not incorporate detailed numeric information such as sample level palaeomagnetic measurements or detailed derived information such as palaeosecular variation data. Both PALEOMAGIA and PINT(_QPI_) use Digital Object Identifiers (DOIs) to provide access to corresponding entries in MagIC, where in-depth palaeomagnetic directional and intensity information are available, and the maintainers work in co-operation with the MagIC database team to ensure data integrity. There is also a possibility to extract GMAP format data files^39^ from PALEOMAGIA, and these can be readily used in both GMAP and in the modern Gplates plate tectonic reconstruction software^40^, (http://www.gplates.org/).

Each PALEOMAGIA entry has a unique reference number, which can be used for referring to a specific pole from an external website. In these cases, the URL follows the convention ‘http://www.helsinki.fi/paleomagia/polelist.php#row[x]’, where [x] is the result number of the pole in PALEOMAGIA. Links to relevant poles in the pole list as well as links to PALEOMAGIA age references are also provided at the search result page. Whenever the current edition of PALEOMAGIA, or PINT(_QPI_) as a whole is being cited, the appropriate citation is to the present Scientific Data paper, not to previous papers, which describe older and substantially different versions of these databases^6,22^.

PALEOMAGIA is being updated regularly and the number of the current version is visible at the database website. Users of the PINT database and its modifications, including PINT(_QPI_), are particularly advised to check the version to use, since at the PINT website (http://earth.liv.ac.uk/pint/), various options are available. Currently, PINT data can be printed at the query form page or downloaded as MS Excel XML format (xlsx), yet Q_PI_ quality grades are currently available only for the PINT(_QPI_) data via QPI Wiki and not at the PINT website, which lists all PINT data, including those from the Phanerozoic.

The future prospects of PALEOMAGIA include the inclusion of Cambrian data, which would facilitate the timing and modelling of the Vendian-Cambrian supercontinent Pannotia, or greater Gondwana^41,42^. Database administrators also consider the inclusion of younger geological periods. The PINT(_QPI_) database will be also extended to the Phanerozoic by grading current Phanerozoic palaeointensity data and by adding data from newly published papers. Discussions on linking PALEOMAGIA more closely to EPOS (European Plate Observing System, https://www.epos-ip.org/) have begun in spring 2017.

## Additional Information

Table 3 is only available in the online version of this paper

**How to cite this article:** Veikkolainen, T.H. *et al.* Advancing Precambrian palaeomagnetism with the PALEOMAGIA and PINT(QPI) databases. *Sci. Data* 4:170068 doi: 10.1038/sdata.2017.68 (2017).

**Publisher’s note:** Springer Nature remains neutral with regard to jurisdictional claims in published maps and institutional affiliations.

## Supplementary Material



## Figures and Tables

**Table 1:** Q_PI_ quality criteria used in PINT(_QPI_) database (see also ref. 9).

**Criterion number**	**Criterion name**	**Explanation**
1	AGE	A reliable (if approximate) age exists and palaeomagnetic behaviour is consistent with a palaeointensity derived from a primary component of remanent magnetization.
2	STAT	The site mean is derived from a minimum of 5 individual sample estimates and these have low dispersion (true standard deviation /mean≤25%).
3	TRM	There exists reasonable independent (e.g., microscopic) evidence that the component of remanence in the bulk of samples is likely a thermoremanent magnetization (TRM).
4	ALT	There exists reasonable evidence that the estimate was not significantly biased by alteration occurring during the experiment.
5	MD	There exists reasonable evidence that the estimate was not significantly biased by multidomain behaviour during the experiment.
6	ACN	There exists reasonable evidence that the estimate was not significantly biased by anisotropy of TRM, cooling rate effects, and nonlinear TRM effects.
7	TECH	The estimate comprises a mean of results derived using more than one palaeointensity technique.
8	LITH	The estimate comprises results from more than one lithology or from samples from the same lithology showing significantly different unblocking behaviour of magnetization.
9	MAG	The raw measurement data are freely available in a public database or repository.
Each criterion can have two values (1=yes, 0=no) and the total Q_PI_ value is the sum of individual criteria.		

**Table 2:** Tables and relations in PALEOMAGIA database. In the main data table, RES# (result number) is unique to each row, but not a key because it is not related to properties in other tables.

**Name of table and description of contents**	**Number of rows**/**columns**	**Keys and relations**
*agerefs*References of isotopic and other types of age data (short journal names, authors, DOI, etc.)	608/8	**SHORTREF** (primary) related to **AGEREF** and **AGEREF2** (foreign keys in table *data*)
*countries*Country name abbreviations and full country names.	56/2	**CNTRY** (primary) related to **CNTRY** (foreign key in table *data*)
*data*The main data table of PALEOMAGIA. Includes palaeomagnetic entries associated with their result numbers (RES#).	3,494/37	**AGEREF** (foreign) related to **SHORTREF** (primary key in table *agerefs*)**AGEREF2** (foreign) related to **SHORTREF** (primary key in table *agerefs*)**CNTRY** (foreign) related to **CNTRY** (primary key in table *countries*)**CRAT** (foreign) related to **CRATON** (primary key in table *terranes*)**PMAGREF** (foreign) related to **PMAGREF** (primary key in table *pmagrefs*)
*pmagrefs*References of palaeomagnetic data (short journal names, authors, DOI, etc.).	1,013/9	**PMAGREF** (primary) related to **PMAGREF** (foreign key in table *data*)
*terranes*Terrane names and corresponding present day continents.	104/2	**CRATON** (primary) related to **CRAT** (foreign key in table *data*)
Numbers of rows and columns refer to version 2.03 of the database, as of March 9, 2017.		

**Table 3:** Column structure in the main data table (*data*) of PALEOMAGIA and associated MySQL data types

**column**	**data type**	**explanation**
RES#	varchar	Unique result number.
ROCK	varchar	Type of rock (igneous, sedimentary or metamorphic).
ROCKUNIT	varchar	Name of rock.
CNTRY	varchar	Country name abbreviation. See table *countries* for full names.
COMP	varchar	Component of magnetization vector.
CRAT	varchar	Terrane (craton, orogen, inlier, etc.). See table *terranes* for relations between terranes and present-day continents.
SLAT	decimal	Latitude of the sampling site in degrees.
SLON	decimal	Longitude of the sampling site in degrees.
LMA	int	Estimated lower limit of the age of magnetization in million years.
HMA	int	Estimated upper limit of the age of magnetization in million years.
ISOAGE	varchar	Isotopic age, if available.
AGEREF	varchar	The age reference most relevant to the palaeomagnetic entry. See table *agerefs*for publications or other sources where the age data have been obtained from, including information about authors, title, name of the journal or other medium of publication, year of publication, volume, pages and DOI.
AGEREF2	varchar	A possible second age reference for the palaeomagnetic entry.
MET	varchar	Method of the age determination.
AGE	int	Estimated age of magnetization, by definition between LMA and HMA.
B	varchar	Number of sampling sites.
N	varchar	Number of samples. This is succeeded by an asterisk (*) if sample mean statistics have been used (otherwise site mean statistics are assumed)
P	varchar	Magnetic polarity (normal; N, reversed; R, mixed; M or combination of distinct normal and reversed polarity entries in polarity pairs; C).
R%	varchar	Percentage of reversed magnetic polarity in sample-level data.
D	decimal	Declination in degrees.
I	decimal	Inclination in degrees.
alfa95	decimal	Radius of 95% cone of confidence for direction in degrees.
k	decimal	Concentration parameter of directions.
PLAT	decimal	Latitude of paleomagnetic pole, or antipole, depending on APWP consideration.
PLON	decimal	Longitude of paleomagnetic pole, or antipole, depending on APWP consideration.
DP	decimal	Angular length of the semi-axis of the 95% confidence for pole, along the site-to-pole great circle in degrees.
DM	decimal	Angular length of the semi-axis of the 95% confidence for pole, perpendicular to the site-to-pole great circle in degrees.
1	int	Truncated Van der Voo grading (Q_Voo_) 1: the rock age is well-determined and the magnetization is presumably of the same age.
2	int	Truncated Van der Voo grading (Q_Voo_) 2: number of samples is sufficient (N>24), concentration parameter is large (k≥10) and 95% confidence circle for the mean direction is small (α95≤16.0°).
3	int	Truncated Van der Voo grading (Q_Voo_) 3: demagnetization is adequate and demonstrably includes vector subtraction.
4	int	Truncated Van der Voo grading (Q_Voo_) 4: field tests constrain the age of magnetization.
5	int	Truncated Van der Voo grading (Q_Voo_) 5: structural control and tectonic coherence with terrane involved.
6	int	Truncated Van der Voo grading (Q_Voo_) 6: geomagnetic field reversals are present.
PMAGREF	varchar	Palaeomagnetic reference, i.e., where the palaeomagnetic data have been originally published. See table *pmagrefs* for information about authors, title, name of the journal or other medium of publication, year of publication, volume, pages, DOI and whether the data have undergone peer review (1=yes, 0=no).
COMMENT	varchar	Possible comments about the entry. These may be subjective and unless otherwise noted, reflect only the viewpoint of PALEOMAGIA administrators.
RECALC	int	Whether any reasonable recalculations have been done (i.e., for some specific reason, the data are not shown exactly as published). This is a dichotomic field (1=yes, 0=no).
The result number (RES) is unique to each entry and serves as the primary key of the database. Note that the appearance of the online database query result table, including but not limited to column names, is different from the actual table structure shown here. Information generated by PHP scripts (e.g., AV as a sum of columns 1…6) to appear at the PALEOMAGIA website is not included here, but is thoroughly explained in the online documentation. For column structures of other PALEOMAGIA tables, see Table 4.		

**Table 4:** Column structure in tables other than the main data table (*agerefs, countries, pmagrefs, terranes*) of PALEOMAGIA and associated MySQL data types.

**Table name:** ***agerefs***		
**Column**	**Data type**	**Explanation**
SHORTREF	varchar	Author-year reference in short form.
AU	varchar	Author names, including surnames and initials of first names.
TITLE	varchar	Name of the paper or other type of document where the results have been published.
REF	varchar	Name of the journal or book, if applicable. For journals and proceedings with long names, abbreviations have been used as noted in the online documentation.
YR	int	Year of publication.
V	varchar	Volume of publication.
PP	decimal	Pages.
DOI	decimal	Digital Object Identifier, see http://dx.doi.org
Table name: *countries*		
CNTRY	varchar	Abbreviation of the name of the country, e.g., FI for Finland.
CNTRYLONG	varchar	Full name of the country. In the online documentation, these are printed along with abbreviations.
Table name: *pmagrefs*		
PMAGREF	varchar	Equal to SHORTREF in table *agerefs*, but for palaeomagnetic reference instead of age reference.
AU	varchar	Author names, including surnames and initials of first names.
TITLE	varchar	Name of the paper or other type of document where the results have been published.
REF	varchar	Name of the journal or book, if applicable. For journals and proceedings with long names, abbreviations have been used as noted in the online documentation.
YR	int	Year of publication.
V	varchar	Volume.
PP	varchar	Pages.
REV	int	Whether the data have undergone peer-review. This is a dichotomic field (1=yes, 0=no)
DOI	varchar	Digital Object Identifier, see http://dx.doi.org
Table name: *terranes*		
CRATON	varchar	Precambrian terrane (in most cases craton) name, e.g., Karelia.
CONT	varchar	Present-day continent where the craton is located. e.g., Europe for the Karelia craton.
For column structure of the main data table, see Table 3 (available online only).		

**Table 5:** PINT(_QPI_) database structure.

**Name of table and description of contents**	**Number of rows**/**columns**	**Keys and relations**
*Refs*References of palaeointensity results (short journal names, authors, DOI, etc.)	64/9	**REFNO**(primary) related to **REF** and **REF2** (foreign keys in tables PINT*data and PIMethods*)
*PINTData*The main data table of PINT(_QPI_). Includes palaeointensity entries associated with their result numbers (DATA).	642/39	**DATA**(primary)**REF** (foreign) related to **REFNO** (primary key in table *Refs*)**INTM** (foreign) related to **INTMETH** (primary key in table *PIMethods*)
*PIMethods*A reference table describing palaeointensity method codes and giving publication where method was first applied	25/2	**INTMETH**(primary) related to **INTM** (foreign key in table PINTData)**REF2**(foreign) related to **REFNO** (primary key in table *Refs*) and **REF** (foreign key in tables *PINTData*)
*Information*A reference table describing all fields in *PIMethods*	39/2	All records match uniquely the field codes of table *PINTData.*
